# Optimizing Flexor Digitorum Profundus Tendon Repair: A Narrative Review

**DOI:** 10.3390/jfb16030097

**Published:** 2025-03-11

**Authors:** Rishith R. Mereddy, Emily E. Zona, Camille J. LaLiberte, Aaron M. Dingle

**Affiliations:** Division of Plastic and Reconstructive Surgery, University of Wisconsin School of Medicine and Public Health, Madison, WI 53792, USA; mereddy@wisc.edu (R.R.M.); ezona@wisc.edu (E.E.Z.); cjlaliberte@wisc.edu (C.J.L.)

**Keywords:** flexor tendon repair, biomaterials for tendon repair, nanomaterials for tendon repair, zone II flexor tendon repair

## Abstract

Zone II flexor digitorum profundus (FDP) tendon injuries are complex, and present significant challenges in hand surgery, due to the need to balance strength and flexibility during repair. Traditional suture techniques often lead to complications such as adhesions or tendon rupture, prompting the exploration of novel strategies to improve outcomes. This review investigates the use of flexor digitorum superficialis (FDS) tendon autografts to reinforce FDP repairs, alongside the integration of biomaterials to enhance mechanical strength without sacrificing FDS tissue. Key biomaterials, including collagen–polycaprolactone (PCL) composites, are evaluated for their biocompatibility, mechanical integrity, and controlled degradation properties. Collagen-PCL emerges as a leading candidate, offering the potential to reduce adhesions and promote tendon healing. Although nanomaterials such as nanofibers and nanoparticles show promise in preventing adhesions and supporting cellular proliferation, their application remains limited by manufacturing challenges. By combining advanced repair techniques with biomaterials like collagen-PCL, this approach aims to improve surgical outcomes and minimize complications. Future research will focus on validating these findings in biological models, assessing tendon healing through imaging, and comparing the cost-effectiveness of biomaterial-enhanced repairs with traditional methods. This review underscores the potential for biomaterial-based approaches to transform FDP tendon repair.

## 1. Introduction

**Figure 1:**
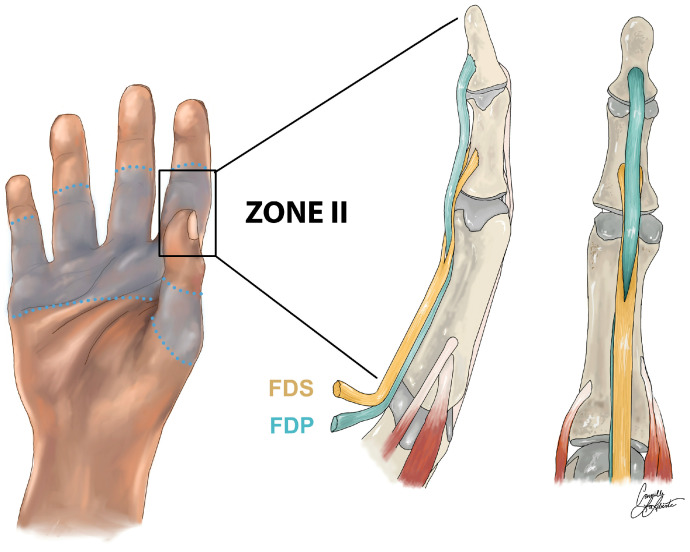
An illustration of the anatomy of zone II flexor tendons.

Zone II flexor digitorum profundus (FDP) tendon injuries are a cause of significant morbidity, and occur at an approximate rate of 33.2 per 100,000, with a notable 43% of these being zone II flexor tendon injuries [1]. The FDP is essential for enabling flexion of the distal interphalangeal joint, the joint closest to the fingertip of each finger (Figure 1), playing a vital role in the fine motor functions of the hand [2]. Since the first flexor tendon repair was reported in 1917, a plethora of repair techniques and models have been suggested [3]. More than a century later, there still remains no consensus on how best to treat flexor tendon injuries. In fact, prior to 1995, most methods were the result of unsupported trial-and-error [4]. Over the past three decades, research has focused on applying more and more core sutures, in an effort to increase the load-bearing capacity of tendons during the healing phase [5,6]. Despite these efforts, the primary complications of tendon repair remain, which are rupture at the repair site and adhesions of the regenerating tendon to the surrounding tissues. These two complicating factors are in direct juxtaposition [5,6]. Zone II flexor tendon injuries are complex, requiring a balance between the strength of the repair and the physical bulk of the repair [7]. Greater bulk leads to significant adhesion, and therefore less range of motion for the patient, while less bulk allows for improved range of motion, but the sutures are more likely to rupture [3]. The complexity of FDP tendon injuries necessitates innovative materials and techniques for successful repair. The majority of FDP repairs consist of a technically difficult procedure involving suture techniques ranging from two to six epitendinous sutures [6,8]. Another challenge associated with this repair is the significant risk of rupture during post-operative rehabilitation therapy [9]. It is estimated that the incidence of suture rupture is significantly greater for zone II FDP injuries compared with injuries in zones I, III, IV, and V, with a relative distribution of 43% for zone II, compared to 29% for the next-closest zone (III) [1]. Early mobilization of the affected finger is critical for a successful functional outcome, as movement promotes tendon healing, increases tensile strength, and decreases adhesion formation [8,10]. However, this active motion increases the risk of suture/tendon rupture. To meliorate post-operative rupture, innovation in FDP repair methods has revolved around suture techniques that increase strength, while limiting the bulk of the repair. For a comprehensive evaluation of these suture techniques, see the recent systematic review [6].

Advances in flexor tendon repair have recently focused on the development of new biomaterial-based strategies, rather than traditional suture techniques. For instance, Reisdorf et al. demonstrated that extrasynovial tendon graft coating with carbodiimide-derivatized synovial fluid and cd-SF-gel reduced adhesion formation and improved long-term functional outcomes in a canine flexor tendon reconstruction model [11]. Similarly, Yaşar discovered that encircling the in zone II tendon repair site with a collagen sheet may act as an effective anti-adhesion barrier, leading to possibly improved tendon mobility in a clinical setting [12]. In another hybrid approach, Chen et al. created a nanoyarn scaffold mixed with tenogenic adipose-derived stem cell sheets, which promoted improved tendon regeneration via cellular alignment and extracellular matrix deposition [13]. Together, these studies demonstrate the potential of integrating biomaterial modification and tissue engineering strategies to overcome the drawbacks of conventional tendon repair.

**Figure 2:**
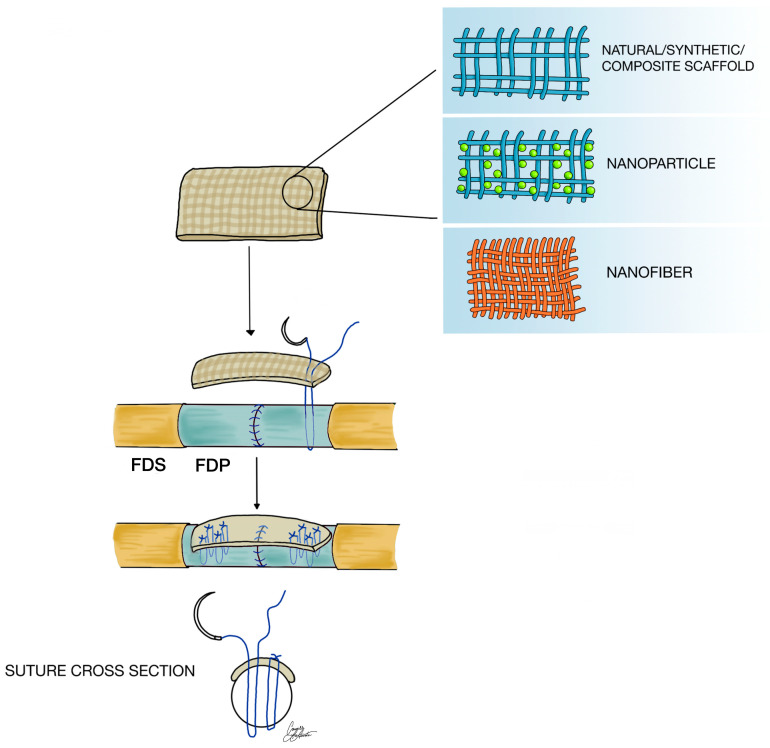
An illustration of the novel flexor tendon repair technique, with the use of various scaffold types. Arrows indicate the chronological order of scaffold application.

While these studies underscore the potential of biomaterial modifications and advanced scaffold strategies to enhance tendon repair, alternative approaches continue to be pursued to further improve clinical outcomes. In an attempt to move away from the classic suture-based repair of the FDP, Zeng et al. utilized a portion of flexor digitorum superficialis (FDS) tendon autograft to buttress the FDP tendon repair site (Figure 2), distributing the tensile forces and ultimately leading to a lower incidence of post-operative suture failure in cadaveric testing [10]. Nevertheless, harvesting the necessary part of the FDS tendon poses a challenge, as donor tendon is not always available. In the last decade, it has become more common to repair the FDS where possible; however, leaving the FDS unrepaired may not impact functional outcomes. Either way, the incorporation of biomaterial-based tendon buttresses as alternatives to scavenged FDS tissue arises as a compelling solution (Figure 2). By capitalizing on the unique properties of these materials, such as their capacity to deliver drugs or extracellular matrix components, we can facilitate accelerated tendon regrowth and ensure optimal alignment, therefore offering a more efficient and superior approach to the healing process.

This narrative review investigates the application of biomaterials and/or nanomaterials in FDP tendon graft repair, utilizing Zeng et al.’s method [10], with a focus on their biomechanical properties, biocompatibility, degradation rates, and potential for improving tendon healing.

### 1.1. Biomaterials and Nanomaterials

#### 1.1.1. Definition and Significance

Biomaterials are substances that are compatible with biological systems, and they aim to treat, augment, or replace various tissues, organs, or functions of the body [14]. Nanomaterials operate at the nanoscale, offering capabilities such as targeted drug delivery, and improve the mechanical strength of intracellular scaffolds used in tissue engineering [14].

#### 1.1.2. Application in Tendon Repair

In the realm of tendon repair, the integration of biomaterials and nanomaterials holds immense promise for enhancing the impact and effectiveness of surgical interventions. Biomaterials, such as collagen and silk fibroin, mimic the extracellular matrix, enhancing cell adhesion and healing, while nanomaterials allow for targeted drug delivery and the enhancement of mechanical properties [15,16,17,18]. Advanced fabrication techniques, such as electrospinning, further optimize these materials for more effective and minimally invasive tendon repair methods [19].

## 2. Materials and Methods

PubMed was used to query for articles. The search criteria specified that included articles were systematic reviews published after 2020. The three search terms used were the following: “biomaterials for tendon repair”, “scaffolds for tendon repair”, and “nanomaterials for tendon repair”. Eight studies met our initial search criteria, but five were removed due to lack of applicability to flexor tendon repair. Due to the lack of studies involving nanomaterials, the literary review from Parchi et al., which describes nanoparticles and nanomaterials for tendon healing, was chosen to address this gap [20]. The three systematic reviews and one literary review were summarized (Table 1).

These reviews provide different perspectives on biomaterials and nanomaterials for tendon repair, and given that the majority were systematic in nature, we elected to write this narrative review as a means of integrating them into one concise summary. Tang et al. reviewed functional biomaterials for the repair of tendons in various animal models, such as humans, rabbits, rat, and sheep [14]. In their observations, functional biomaterials have the potential to imitate both mechanical and biological properties of tendons, especially in the case of complex injuries like supraspinatus tears [14]. Zhang et al. focused on silk scaffolds, underlining their mechanical strength and biocompatibility, especially in the repair of Achilles tendons, which further supports their possible applicability for FDP tendon repair [21]. Mao et al. researched engineered scaffolds, and showed their adaptability in models ranging from the long digital extensor to the supraspinatus [22]. The results further underlined that scaffold properties should be tailored to the specific tendon type for optimal healing. Parchi et al. gave an overview of nanomaterials and nanoparticles, outlining their advantages in drug delivery and biological interaction, although their application remains limited due to manufacturing challenges [20]. Altogether, these studies, with the supplementation of original and recent works in the field, emphasize how material properties and scaffold design are important in the development of new tendon repair techniques.

## 3. Material Properties

### 3.1. Nanomaterials

Nanomaterials offer unique advantages due to their size. For example, their nanoscale allows for precise control over the surface characteristics of the material, which can be utilized to enhance cell adhesion and proliferation [20,22,23]. Recent innovations have focused on developing nanofibers and nanoparticles that can be incorporated into engineered scaffolds [20,22,24]. These recent innovations underscore the potential of nanofiber- and nanoparticle-based treatments in tendon repair. The focal point is exploiting the scale of nanomaterials to enhance mechanical strength and stimulate positive biological interactions that are vital for the regeneration of tendon tissue [23]. The unique properties of nanomaterials, including their ability to be heavily customized, have been harnessed to create scaffolds that not only replicate the natural tendon environment, but also improve cell adhesion and proliferation (Figure 2) [20].

There are two primary types of nanomaterials used in tendon repair, each offering specific advantages. Nanofibers, for instance, can be used to provide a structurally intricate scaffold that mimics the collagen fibers in native tendons. This mimicry at the nanoscale level facilitates improved cell interactions and tissue integration [17]. Nanoparticles, on the other hand, allow for controlled drug delivery and electrical stimulation, which may add to the overall effectiveness of the scaffold and possibly enhance tendon healing [20].

### 3.2. Natural Materials

#### 3.2.1. Silk Fibroin and Natural Silk

Silk fibroin (SF) emerges as a compelling natural material with a history of use in surgical care. It was initially recognized for its clinical utility as a suture material. In the realm of tissue engineering (TE), SF has been used due to its useful characteristics, such as toughness, biocompatibility, biodegradability, low immunogenicity, and thermal stability [21]. The mechanical and porosity properties of SF can be customized, depending on the clinical application, by adjusting SF solution concentrations [21]. This versatility allows for precise customization, facilitating the alteration of biodegradability by modifying the B-sheet structure of the SF scaffold [25]. Biodegradability is a critical aspect of tendon repair as a whole, and SF excels by supporting cell proliferation, while undergoing gradual degradation, over several weeks to months, into nontoxic chemicals, aligning with the body’s healing processes and lowering the chance of complications after the scaffold is implanted [26]. SF can be engineered into many mediums, including films, hydrogels, cords, knitted structures, electrospun fibers, composite scaffolds, and particles [21,26]. While SF-based biomaterials exhibit inherently weaker mechanical properties, natural silk can function as an alternative, due to its stronger hierarchical structure than SF.

#### 3.2.2. Collagen

Collagen, a major component of natural tendon tissue, stands as a fundamental natural material in the realm of tendon repair [27]. Recognized for its remarkable biocompatibility and its role as the predominant fiber in tendons, collagen takes center stage in engineered extracellular matrix (ECM) applications [21,28]. A drawback associated with collagen scaffolds is their lack of mechanical strength, due to the inherent weakness of the material; however, when strategically arranged in a parallel manner, collagen harnesses its biocompatibility to maximize mechanical strength, aligning seamlessly with the stringent requirements of tendon repair [22]. Collagen scaffolds also have excellent biodegradability, but this rapid degradation may not always align with the time frame associated with post-operative recovery. Despite the limitations of the material, the good binding sites, for cells and growth factors, of collagen makes its use in FDP tendon repair more promising [14,29].

### 3.3. Synthetic Materials

Synthetic materials, including polylactic acid (PLA), polyglycolic acid (PGA), polylactic-co-glycolic acid (PLGA), and polycaprolactone (PCL), provide greater control over mechanical properties and degradation rates [22,30]. PLA and PGA, both biodegradable polymers, have been extensively used in medical applications, due to their strength and predictable degradation profiles [30,31]. PCL, known for its slow degradation rate, offers prolonged mechanical support, making it suitable for tendon repair applications where extended healing time is required [8,31]. The primary issue with synthetic polymers (PLA, PGA, PCL) is their dense structure with significantly less porosity than that of their natural counterparts [9]. With this in mind, for greater mechanical properties, cell proliferation and adhesion are sacrificed when opting for synthetic over natural biomaterials. The use of polymers such as PLGA, a copolymer of PLA and PGA, may be a solution to the sacrifice of cell proliferation and adhesion that comes with the use of more common synthetic polymers. PLGA can be loaded with growth factors or genetic material to promote growth, due to its linear aliphatic structure (Figure 2) [9]. Loading growth factors may mitigate the materials’ limitations to cell proliferation, including angiogenesis, and can possibly help to control the inflammation and host response that comes with all biomaterials.

### 3.4. Composite Materials

Composite materials, combining natural and synthetic components, offer a synergistic approach to tendon repair [32]. By blending materials like collagen with synthetic polymers such as PCL, composites can achieve the necessary balance between biocompatibility, mechanical strength, and degradation rate [33]. This category of materials is particularly promising for FDP tendon repair, due to its ability to provide immediate mechanical support, while promoting cellular activities for long-term tissue regeneration [33]. A customized blend of materials can be made to suit the needs of the particular tendon repair [34]. One anticipated barrier to the success of this method may be the resource-intensive nature of manufacturing these composite materials.

## 4. Methods of Biomaterial Scaffold Fabrication

The fabrication of scaffolds for tendon repair employs a range of techniques, each suited to creating structures that support the complex biological and mechanical needs of tendon healing. These methods enable the precise engineering of scaffold architecture, porosity, and biochemical properties, crucial for mimicking the natural tendon environment and promoting effective tissue integration and regeneration.

### 4.1. Electrospinning

Electrospinning is an adaptable fabrication technique used to create fibrous scaffolds with sizes on both the microscale and nanoscale, depending on the user’s needs [9]. This method is particularly effective for producing nanofibers from both synthetic and natural polymers, allowing for the creation of scaffolds that closely mimic the extracellular matrix of tendons [14,19]. Electrospun scaffolds can be engineered to have a high porosity and large surface area, enhancing cell adhesion, proliferation, and differentiation [35]. By adjusting parameters such as polymer concentration, voltage, and spinning distance, the mechanical properties and degradation rates of the fibers can be finely tuned [21].

### 4.2. Three-Dimensional Printing

Three-dimensional printing, or additive manufacturing, offers unparalleled precision in designing scaffold structures with complex geometries and tailored mechanical properties [36]. This technique can fabricate scaffolds layer by layer, using materials ranging from biodegradable polymers to bioceramics [21]. The ability to control the scaffold’s macro- and micro-architecture enables the creation of constructs that not only support mechanical loads, but also facilitate vascularization and nutrient diffusion, which are essential for tendon repair and regeneration [37].

### 4.3. Knitting and Weaving

Knitting and weaving are traditional textile fabrication techniques that have been adapted for biomedical applications to produce scaffolds with specific, tailored mechanical properties and porosities [21,38]. These methods are particularly suitable for creating large, structurally robust constructs that can withstand the mechanical demands of tendon repair [39]. Knitted and woven scaffolds offer the advantage of being able to mimic the anisotropic mechanical properties of native tendons, providing directional strength and flexibility that supports the healing process [21].

### 4.4. Embroidery

Embroidery, a classical textile fabrication technique, has been adapted in tissue engineering to fabricate scaffolds that replicate the complex architecture and mechanical behavior of native tendons. The technique involves the precise stitching of biocompatible yarns, such as PLA or poly(lactic-co-ε-caprolactone), into preprogrammed patterns that replicate the hierarchical architecture of tendon tissue [40]. The embroidery technique gives precise control over fiber directionality, packing density, and overall scaffold geometry, which are critical factors influencing cell alignment, growth, and differentiation during tendon repair [41].

Preparation begins with the selection of appropriate thread materials offering the desired mechanical properties and biocompatibility. The threads are embroidered onto a substrate using a pre-designed pattern that replicates the anisotropy of tendons [40,41,42]. Highly advanced embroidery machines enable the manufacture of complex, three-dimensional scaffold architectures with great reproducibility [41]. Scaffolds can undergo surface treatments or coatings after embroidery to enhance cell adhesion and bioactivity. For instance, coating embroidered scaffolds with extracellular matrix materials like collagen enables further cellular integration and tissue regeneration [42].

Experiments have demonstrated that embroidered scaffolds provide not only mechanical support, but also facilitate organized extracellular matrix deposition by inducing cell orientation [40]. This biomimetic approach ensures that the newly formed tissue closely resembles the native tendon in structure as well as function, thereby improving the efficacy of tendon repair strategies [43].

### 4.5. Hybrid Techniques

Hybrid fabrication methods combine two or more techniques to leverage the unique advantages of each, creating composite scaffolds that offer optimal support for tendon repair [8]. For instance, electrospun nanofibers can be layered or embedded within 3D-printed structures to add microscale texture that enhances cell attachment and proliferation [8,44]. Similarly, knitting or weaving can be used to create the scaffold’s macrostructure, with electrospinning or 3D printing adding microscale features that guide cellular behavior [44].

The choice of fabrication method depends on the specific requirements of the tendon repair, including the need for mechanical strength, biocompatibility, and the promotion of cellular activities for tissue regeneration. By carefully selecting and combining materials and fabrication techniques, researchers and clinicians can create scaffolds that significantly improve the outcomes of FDP tendon repairs, offering patients faster recovery times and a reduced risk of complications [14].

## 5. Biomaterials and Nanomaterials in Tendon Repair

A comparative analysis of the various biomaterials and nanomaterials covered in the literature for application in FDP tendon repair was summarized (Table 2). Table 2 organizes the materials, along with their corresponding characteristics, including biocompatibility, mechanical strength, degradation rate, integrability with common suture techniques, and effectiveness in healing.

## 6. Discussion

### 6.1. Application in FDP Tendon Repair

#### 6.1.1. Nanomaterials and Nanoparticles

Nanofibers and nanoparticles show great promise in enhancing the FDP tendon repair process. As discussed previously, nanofiber scaffolds have been shown to improve cell adhesion and proliferation, which are important facets of tendon healing [14,20,23]. Nanoparticles allow for the ability to deliver drugs, which can lead to reduced inflammation at the repair site and possibly improved tissue regeneration [20,24]. Nanoparticles have the ability to locally deliver anti-inflammatory agents and other factors to the repair site to facilitate improved regeneration. These nanomaterials can be used in combination with traditional sutures, or integrated into bioactive scaffolds that hold the repair together. Nanomaterials can provide less bulk during the FDP repair, allowing for greater mobility post-operation [20]. The minimal bulk is especially beneficial in FDP tendon repair, as it will improve motility post-operatively and reduce the risk of adhesion [10]. Furthermore, the highly customizable nature of nanomaterials as a whole allows for the tailoring of their properties to meet specific clinical needs. Nanomaterials may be incorporated into sutures to enhance their mechanical strength and promote the healing process.

While this is appealing conceptually, integrating these materials with traditional suture techniques may prove to be complex. Additionally, the weaker mechanical properties of nanomaterials, particularly in load-bearing applications, necessitate advanced tissue engineering techniques to allow for their usage in tendon repair [17,20,22]. These approaches may include combining these nanomaterials and nanoparticles with common biomaterial scaffolds. Current use cases of nanomaterials and nanoparticles are limited in FDP tendon repair, but there have been remarkable advances recently in the use of nanoscale engineering for FDP tendon repair. Examples include the previously mentioned nanoyarn scaffold mixed with tenogenic adipose-derived stem cell sheets, which has shown potential for tendon repair in rabbit models, and a novel electrospun water-borne polyurethane nanofibrous membrane, which has the potential to reduce the degree of peritendinous adhesion without any added cytotoxicity [13,45]. There is clear potential for the use of nanomaterials in FDP tendon repair, and with continued advancements, they may revolutionize FDP tendon repair.

#### 6.1.2. Natural Materials

Collagen- and silk-based biomaterials are primary options when it comes to natural materials for FDP tendon repair. Their excellent biocompatibility and ability to function as an analog to the tendon’s ECM may allow for their integration into FDP tendon repair [27]. Collagen, a key structural protein in native tendons, provides a biologically favorable environment for cell growth and adhesion [21,25,27]. Although tendons are composed of collagen, scaffolds made of collagen are normally too mechanically weak to withstand rehabilitation post-operatively, leading to both scaffold rupture and tendon rupture [21]. This may be solved by using more collagen, but in FDP repair, space is very important, and being able to use less material is always favored [10]. In recent studies, it has been indicated that aligning collagen fibers within the scaffold allows for improved mechanical properties and cellular interaction. However, this is not to say that collagen has no use in tendon repair; recent studies utilizing collagen have shown its strength in preventing adhesions at tendon repair sites [46,47]. Collagen can be used at the repair site to support tendon healing as an adjunct to suture techniques. Despite collagen’s benefits, it also suffers from rapid degradation. The issues regarding mechanical properties and degradation require further engineering to solve.

Silk, therefore, becomes particularly intriguing, given tendons’ high tensile strength [21,26]. The versatility of SF allows for the consideration of knitted, composite, and cord scaffolds, each addressing the mechanical challenge in repair, while simulating the original tendon shape [21,25,26]. Silk presents itself as a strong candidate for use in FDP repair. Studies have also pointed out the potential of non-mulberry silk, which may elicit more favorable immune responses, thus offering a very exciting avenue for future exploration [21]. However, the extraction and processing of high-quality silk fibroin remain expensive and complicated, presenting a significant barrier to its widespread clinical use [21,26].

Improvements in fabrication techniques, including electrospinning and 3D printing, hold promise in the future of optimizing the application of both collagen- and silk-based biomaterials [25]. Moreover, the rise in popularity of 3D bioprinting opens new avenues for the creation of scaffolds not only from collagen and silk, but also from alginate, chitosan, and more [48]. Through the refinement of these techniques, and by addressing present challenges, we may enable collagen- and silk-based biomaterials to change the face of FDP tendon repair, and provide a more long-lasting effective solution for patients.

#### 6.1.3. Synthetic Materials

Synthetic materials such as PGA, PLA, and PCL offer great mechanical strength, with the ability to control degradation rates [14,22,30]. These materials provide the capacity to exert control over the healing process, and may exhibit equal or greater strength when compared to a traditional tissue graft, two features which are likely to improve clinical outcomes [14,30]. These materials can be used in the form of braided sutures or scaffold meshes that are sutured into the tendon gap to provide initial strength during the healing process. Due to their mechanical strength, less total material can be used to buttress the FDP repair, minimizing the issue of bulk without compromising the mechanical properties. Additionally, the degradation rates of these polymers can be tailored to align with the natural healing timeline, ensuring a gradual transfer of load to regenerating tissue [14,22]. Surgical techniques may involve placing these synthetic materials around the repaired tendon, where they gradually degrade and are replaced by new tendon tissue. However, challenges exist in the optimization of their porosity and surface properties to facilitate cell proliferation and integration [22]. Strategies such as surface modifications, copolymerization with natural materials, or incorporating bioactive agents can be used to address these challenges [22,34]. These features and innovations underscore the potential for synthetic polymers to be utilized in advancing FDP tendon repair.

#### 6.1.4. Hybrid Materials

Hybrid materials such as collagen–PCL offer an alternative to traditional natural or synthetic biomaterials [9,34]. Hybrid materials combine the excellent biocompatibility of natural materials with the mechanical strength of synthetic materials [22]. This combination provides a balanced approach to tendon repair, achieving sufficient mechanical strength without compromising the biological environment [14,22,32]. In FDP tendon repairs, hybrid scaffolds are commonly used to reinforce tendon repair sites, where they serve as both a biologically favorable environment and a structurally supportive scaffold for healing. Hybrid materials also support various manufacturing techniques, such as electrospinning, 3D printing, and knitting, allowing for the creation of scaffolds with tailored properties to meet the specific needs of FDP tendon repair [19,21,22,36]. Moreover, the ability to incorporate growth factors and bioactive molecules in the scaffold enhances the potential of cellular proliferation and tendon regeneration [14,22]. Despite having the most potential for tendon repair, the high cost and limited availability of manufacturing equipment remain barriers to the widespread usage of hybrid materials [32]. The promise of these materials highlights the need for further research to optimize production for their large-scale use in clinical environments.

### 6.2. Best Material for FDP Tendon Repair

Based on the results of the comparative analysis, collagen–PCL displays itself as the best option for application in FDP tendon repair, utilizing Zeng et al.’s method (Table 2) [10]. This conclusion is drawn from its optimal balance of mechanical integrity, biocompatibility, and support of the healing process.

## 7. Future Directions

This review highlights the potential of biomaterial-based interventions for FDP tendon repair. Although promising, there remains a significant gap in the literature regarding the real-world effectiveness of these interventions, particularly in zone II flexor tendon injuries. Moving forward, there exist several key areas on which future research should focus.

Preliminary studies could begin with the testing of these various biomaterial interventions in cadaver models, to understand their biomechanical properties and to refine the materials and methods, before moving onto in vivo studies. Following the cadaver studies, animal models could examine the longitudinal outcomes of these interventions, while also analyzing the biological mechanisms through which these materials promote healing and affect other cells at the molecular level. Moreover, it is important to investigate the integration of biomaterials with both novel suture techniques and more traditional suture techniques [10,49]. This would help to determine the best combination of suture techniques and material to effectively optimize the intervention for optimal healing outcomes. Finally, evaluating the cost-effectiveness of biomaterial-based interventions compared to traditional methods is important; this analysis should consider both direct medical costs and indirect costs, such as reduced recovery time and improved patient outcomes. In addressing these areas of research, we could gain a more comprehensive understanding of the potential benefits and limitations of biomaterial-based FDP tendon repair, ultimately leading to improved surgical techniques and patient outcomes in the broader field of tendon repair.

By understanding and developing biomaterial-based treatments for FDP tendon repair, we create the foundation for applying these insights to larger tendons, such as the Achilles and larger flexor tendons. Starting with small tendon repairs allows us to refine the biomaterials and techniques in the more precise and smaller environment of the finger. If biomaterial-based treatments prove effective in smaller tendons, the principles and methodologies could be scaled up to address larger tendon injuries. Ultimately, the goal of this work is to develop effective and scalable treatments to improve outcomes for a broader range of tendon injuries.

## 8. Conclusions

Zone II FDP repair represents a persistent clinical challenge, representing opportunities for innovation. This review highlights the potential of biomaterials and nanomaterials to enhance the current state of Zone II FDP tendon repair. These materials offer a balance between mechanical strength and biocompatibility, addressing the challenges of traditional repair methods. Through this review process, we identify collagen–PCL as the leading candidate for enhancing FDP repair, offering the potential to reduce adhesions and promote tendon healing. The application of these materials in both traditional and novel repair techniques may lead to improved patient outcomes. However, while the application of these materials in FDP repair is promising, further research is required. Future studies should focus on both cadaveric and in vivo models, to further refine and verify the materials and techniques best suited to enhancing patient outcomes following Zone II FDP repair, before moving on to clinical trials.

## Figures and Tables

**Table 1:** Included review articles.

Author	Journal	Medical Intervention	Specific Augmentation	Animal Models	Tendon Model
Tang et al. [14]	Regen Biomater	Biomaterial	Functional biomaterials	Human, rabbit, rat, sheep	Infraspinatus, dorsal myofascial, Achilles, supraspinatus tendon, subacromial deltoid bursa in patients with supraspinatus tendon tear
Zhang et al. [21]	ACS Biomater Sci Eng	Silk scaffold	Silk scaffold	Rabbit, rat, sheep	Achilles tendon
Mao et al. [22]	Front Bioeng Biotechnol	Biomaterial	Engineered scaffold	Rabbit, rat	Achilles, supraspinatus, infraspinatus, long digital extensor
Parchi et al. [20] (literary review)	FrontAging Neurosci	Nanomaterial	Nanomaterials	N/A	N/A

**Table 2:** Summary of biomaterials and nanomaterials used in tendon repair.

Material	Biocompatibility	Mechanical Strength	Degradation Rate	Integration with Suture Techniques	Effectiveness in Healing	Source
Nanofibers	High	Moderate	Controllable	Challenging	High	[20]
Nanoparticles	High	Low	Controllable	Moderate	High	[20]
Collagen	High	Low	Rapid	Moderate	High	[21,22]
Collagen	High	Low	Rapid	Moderate	High	[21,22]
Silk fibroin	High	High	Moderate	Good	High	[21]
PLA	Moderate	High	Predictable	Moderate	High	[8,22]
PGA	Moderate	High	Predictable	Moderate	High	[8,22]
PCL	Moderate	High	Slow	Moderate	High	[8,22]
PLGA	Moderate	High	Controllable	Moderate	High	[8,22]
Collagen–PCL	High	High	Controllable	Good	High	[8,22]

## Data Availability

This manuscript is a narrative review, and does not involve the generation of new datasets. All data supporting the findings and discussions in this review are derived from previously published studies, which have been appropriately cited. Readers can access these datasets and studies through the references provided.

## Abbreviations

The following abbreviations are used in this manuscript:

ECM	Extracellular matrix
FDP	Flexor digitorum profundus
FDS	Flexor digitorum superficialis
PCL	Polycaprolactone
PGA	Polyglycolic acid
PLA	Polylactic acid
PLGA	Polylactic-co-glycolic acid
SF	Silk fibroin
TE	Tissue engineering

## References

[1] Tobler-Ammann B.C., Beckmann-Fries V., Calcagni M., Kämpfen A., Schrepfer L., Vögelin E. (2023). Outcomes of Primary Flexor Tendon Repairs in Zones 2 and 3: A Retrospective Cohort Study. J. Hand Surg. Glob. Online.

[2] Lung B.E., Burns B. (2025). Anatomy, Shoulder and Upper Limb, Hand Flexor Digitorum Profundus Muscle. StatPearls.

[3] Tang J.B., Lalonde D., Harhaus L., Sadek A.F., Moriya K., Pan Z.J. (2022). Flexor Tendon Repair: Recent Changes and Current Methods. J. Hand Surg..

[4] Strickland J.W. (1995). Flexor Tendon Injuries: I. Foundations of Treatment. J. Am. Acad. Orthop. Surg..

[5] Rawson S., Cartmell S., Wong J. (2013). Suture Techniques for Tendon Repair; a Comparative Review. Muscles Ligaments Tendons J..

[6] Liu G., Lv G., Liu F. (2024). Suture Techniques in the Surgical Management of Flexor Tendon, Achilles Tendon and Cruciate Ligament Injuries: A Systematic Review. BMC Musculoskelet. Disord..

[7] Stevens K.A., Caruso J.C., Fallahi A.-K.M., Patiño J.M. (2025). Flexor Tendon Lacerations. StatPearls.

[8] Xu H., Huang X., Guo Z., Zhou H., Jin H., Huang X. (2023). Outcome of Surgical Repair and Rehabilitation of Flexor Tendon Injuries in Zone II of the Hand: Systematic Review and Meta-Analysis. J. Hand Surg..

[9] Tang J.B. (2021). Rehabilitation after Flexor Tendon Repair and Others: A Safe and Efficient Protocol. J. Hand Surg..

[10] Zeng W., Albano N.J., Sanchez R.J., Mccabe R., Vanderby R., Poore S.O., Dingle A.M. (2020). Beyond the Core Suture: A New Approach to Tendon Repair. Plast. Reconstr. Surg. Glob. Open.

[11] Reisdorf R.L., Liu H., Bi C., Vrieze A.M., Moran S.L., Amadio P.C., Zhao C. (2023). Carbodiimide-Derivatized Synovial Fluid for Tendon Graft Coating Improves Long-Term Functional Outcomes of Flexor Tendon Reconstruction. Plast. Reconstr. Surg..

[12] Yaşar B. (2023). Encircling Tendon Repair Site with Collagen Sheet in Flexor Zone 2: Retrospective Study. J. Orthop. Surg..

[13] Chen S., Wang J., Chen Y., Mo X., Fan C. (2021). Tenogenic Adipose-Derived Stem Cell Sheets with Nanoyarn Scaffolds for Tendon Regeneration. Mater. Sci. Eng. C Mater. Biol. Appl..

[14] Tang Y., Wang Z., Xiang L., Zhao Z., Cui W. (2022). Functional Biomaterials for Tendon/Ligament Repair and Regeneration. Regen. Biomater..

[15] Hou J., Yang R., Vuong I., Li F., Kong J., Mao H.-Q. (2021). Biomaterials Strategies to Balance Inflammation and Tenogenesis for Tendon Repair. Acta Biomater..

[16] Kim S.E., Kim J.G., Park K. (2019). Biomaterials for the Treatment of Tendon Injury. Tissue Eng. Regen. Med..

[17] Zhang M., Song W., Tang Y., Xu X., Huang Y., Yu D. (2022). Polymer-Based Nanofiber-Nanoparticle Hybrids and Their Medical Applications. Polymers.

[18] Joudeh N., Linke D. (2022). Nanoparticle Classification, Physicochemical Properties, Characterization, and Applications: A Comprehensive Review for Biologists. J. Nanobiotechnol..

[19] Jun I., Han H.-S., Edwards J.R., Jeon H. (2018). Electrospun Fibrous Scaffolds for Tissue Engineering: Viewpoints on Architecture and Fabrication. Int. J. Mol. Sci..

[20] Parchi P.D., Vittorio O., Andreani L., Battistini P., Piolanti N., Marchetti S., Poggetti A., Lisanti M. (2016). Nanoparticles for Tendon Healing and Regeneration: Literature Review. Front. Aging Neurosci..

[21] Zhang L., Zhang W., Hu Y., Fei Y., Liu H., Huang Z., Wang C., Ruan D., Heng B.C., Chen W. (2021). Systematic Review of Silk Scaffolds in Musculoskeletal Tissue Engineering Applications in the Recent Decade. ACS Biomater. Sci. Eng..

[22] Mao Z., Fan B., Wang X., Huang X., Guan J., Sun Z., Xu B., Yang M., Chen Z., Jiang D. (2021). A Systematic Review of Tissue Engineering Scaffold in Tendon Bone Healing in Vivo. Front. Bioeng. Biotechnol..

[23] Hasan A., Morshed M., Memic A., Hassan S., Webster T.J., Marei H.E.-S. (2018). Nanoparticles in Tissue Engineering: Applications, Challenges and Prospects. Int. J. Nanomed..

[24] Adjei-Sowah E., Chandrasiri I., Xiao B., Liu Y., Ackerman J.E., Soto C., Nichols A.E.C., Nolan K., Benoit D.S.W., Loiselle A.E. (2023). Development of a Nanoparticle-Based Tendon-Targeting Drug Delivery System to Pharmacologically Modulate Tendon Healing. BioRxiv Prepr. Serv. Biol..

[25] Qian S., Wang Z., Zheng Z., Ran J., Zhu J., Chen W. (2019). A Collagen and Silk Scaffold for Improved Healing of the Tendon and Bone Interface in a Rabbit Model. Med. Sci. Monit. Int. Med. J. Exp. Clin. Res..

[26] Sun W., Gregory D.A., Tomeh M.A., Zhao X. (2021). Silk Fibroin as a Functional Biomaterial for Tissue Engineering. Int. J. Mol. Sci..

[27] Liu S.H., Yang R.S., al-Shaikh R., Lane J.M. (1995). Collagen in Tendon, Ligament, and Bone Healing. A Current Review. Clin. Orthop..

[28] Sorushanova A., Delgado L.M., Wu Z., Shologu N., Kshirsagar A., Raghunath R., Mullen A.M., Bayon Y., Pandit A., Raghunath M. (2019). The Collagen Suprafamily: From Biosynthesis to Advanced Biomaterial Development. Adv. Mater..

[29] Bhavsar D., Shettko D., Tenenhaus M. (2010). Encircling the Tendon Repair Site with Collagen-GAG Reduces the Formation of Postoperative Tendon Adhesions in a Chicken Flexor Tendon Model. J. Surg. Res..

[30] DeStefano V., Khan S., Tabada A. (2020). Applications of PLA in Modern Medicine. Eng. Regen..

[31] Zhang J., Wang W., Zhang X., Yang L., Zhang J. (2022). Research Progress of Biodegradable Polymers in Repairing Achilles Tendon Injury. Front. Mater..

[32] Gomez-Romero P., Pokhriyal A., Rueda-García D., Bengoa L.N., González-Gil R.M. (2024). Hybrid Materials: A Metareview. Chem. Mater. Publ. Am. Chem. Soc..

[33] Arevalo A., Rao S., Willier D.P., Schrock C.I., Erickson B.J., Jack R.A., Cohen S.B., Ciccotti M.G. (2023). Surgical Techniques and Clinical Outcomes for Medial Epicondylitis: A Systematic Review. Am. J. Sports Med..

[34] Xie Y., Zhang F., Akkus O., King M.W. (2022). A Collagen/PLA Hybrid Scaffold Supports Tendon-Derived Cell Growth for Tendon Repair and Regeneration. J. Biomed. Mater. Res. B Appl. Biomater..

[35] Barcena A.J.R., Mishra A., Bolinas D.K.M., Martin B.M., Melancon M.P. (2024). Integration of Electrospun Scaffolds and Biological Polymers for Enhancing the Delivery and Efficacy of Mesenchymal Stem/Stromal Cell Therapies. Front. Biosci. Landmark Ed..

[36] Do A.-V., Khorsand B., Geary S.M., Salem A.K. (2015). 3D Printing of Scaffolds for Tissue Regeneration Applications. Adv. Healthc. Mater..

[37] Matai I., Kaur G., Seyedsalehi A., McClinton A., Laurencin C.T. (2020). Progress in 3D Bioprinting Technology for Tissue/Organ Regenerative Engineering. Biomaterials.

[38] Rinoldi C., Kijeńska-Gawrońska E., Khademhosseini A., Tamayol A., Swieszkowski W. (2021). Fibrous Systems as Potential Solutions for Tendon and Ligament Repair, Healing, and Regeneration. Adv. Healthc. Mater..

[39] Akbari M., Tamayol A., Bagherifard S., Serex L., Mostafalu P., Faramarzi N., Mohammadi M.H., Khademhosseini A. (2016). Textile Technologies and Tissue Engineering: A Path Toward Organ Weaving. Adv. Healthc. Mater..

[40] Hahn J., Schulze-Tanzil G., Schröpfer M., Meyer M., Gögele C., Hoyer M., Spickenheuer A., Heinrich G., Breier A. (2019). Viscoelastic Behavior of Embroidered Scaffolds for ACL Tissue Engineering Made of PLA and P(LA-CL) After In Vitro Degradation. Int. J. Mol. Sci..

[41] Gögele C., Hahn J., Elschner C., Breier A., Schröpfer M., Prade I., Meyer M., Schulze-Tanzil G. (2020). Enhanced Growth of Lapine Anterior Cruciate Ligament-Derived Fibroblasts on Scaffolds Embroidered from Poly(l-Lactide-Co-ε-Caprolactone) and Polylactic Acid Threads Functionalized by Fluorination and Hexamethylene Diisocyanate Cross-Linked Collagen Foams. Int. J. Mol. Sci..

[42] von Witzleben M., Hahn J., Richter R.F., de Freitas B., Steyer E., Schütz K., Vater C., Bernhardt A., Elschner C., Gelinsky M. (2024). Tailoring the Pore Design of Embroidered Structures by Melt Electrowriting to Enhance the Cell Alignment in Scaffold-Based Tendon Reconstruction. Biomater. Adv..

[43] Gögele C., Konrad J., Hahn J., Breier A., Schröpfer M., Meyer M., Merkel R., Hoffmann B., Schulze-Tanzil G. (2021). Maintenance of Ligament Homeostasis of Spheroid-Colonized Embroidered and Functionalized Scaffolds after 3D Stretch. Int. J. Mol. Sci..

[44] Sinha R., Cámara-Torres M., Scopece P., Verga Falzacappa E., Patelli A., Moroni L., Mota C. (2021). A Hybrid Additive Manufacturing Platform to Create Bulk and Surface Composition Gradients on Scaffolds for Tissue Regeneration. Nat. Commun..

[45] Chen S.-H., Chou P.-Y., Chen Z.-Y., Lin F.-H. (2019). Electrospun Water-Borne Polyurethane Nanofibrous Membrane as a Barrier for Preventing Postoperative Peritendinous Adhesion. Int. J. Mol. Sci..

[46] Brebels J., Mignon A. (2022). Polymer-Based Constructs for Flexor Tendon Repair: A Review. Polymers.

[47] Veronesi F., Giavaresi G., Bellini D., Casagranda V., Pressato D., Fini M. (2020). Evaluation of a New Collagen-Based Medical Device (ElastiCo®) for the Treatment of Acute Achilles Tendon Injury and Prevention of Peritendinous Adhesions: An in Vitro Biocompatibility and in Vivo Investigation. J. Tissue Eng. Regen. Med..

[48] Benwood C., Chrenek J., Kirsch R.L., Masri N.Z., Richards H., Teetzen K., Willerth S.M. (2021). Natural Biomaterials and Their Use as Bioinks for Printing Tissues. Bioengineering.

[49] Kotwal P.P., Ansari M.T. (2012). Zone 2 Flexor Tendon Injuries: Venturing into the No Man’s Land. Indian J. Orthop..

